# Multi-octave two-color soliton frequency comb in integrated chalcogenide microresonators

**DOI:** 10.1007/s12200-024-00139-x

**Published:** 2024-11-11

**Authors:** Huanjie Cheng, Guosheng Lin, Di Xia, Liyang Luo, Siqi Lu, Changyuan Yu, Bin Zhang

**Affiliations:** 1 Guangdong Provincial Key Laboratory of Optoelectronic Information Processing Chips and Systems, School of Electrical and Information Technology, Sun Yat-sen University, Guangzhou, 510275 China; 2 Photonics Research Center, Department of Electronic and Information Engineering, The Hong Kong Polytechnic University, Kowloon, Hong Kong China

**Keywords:** Mid-infrared, Kerr microcombs, Two-color soliton, Multi-octave, Chalcogenide glasses

## Abstract

Mid-infrared (MIR) Kerr microcombs are of significant interest for portable dual-comb spectroscopy and precision molecular sensing due to strong molecular vibrational absorption in the MIR band. However, achieving a compact, octave-spanning MIR Kerr microcomb remains a challenge due to the lack of suitable MIR photonic materials for the core and cladding of integrated devices and appropriate MIR continuous-wave (CW) pump lasers. Here, we propose a novel slot concentric dual-ring (SCDR) microresonator based on an integrated chalcogenide glass chip, which offers excellent transmission performance and flexible dispersion engineering in the MIR band. This device achieves both phase-matching and group velocity matching in two separated anomalous dispersion regions, enabling phase-locked, two-color solitons in the MIR region with a commercial 2-μm CW laser as the pump source. Moreover, the spectral locking of the two-color soliton enhances pump wavelength selectivity, providing precise control over soliton dynamics. By leveraging the dispersion characteristics of the SCDR microresonator, we have demonstrated a multi-octave-spanning, two-color soliton microcomb, covering a spectral range from 1156.07 to 5054.95 nm (200 THz) at a −40 dB level, highlighting the versatility and broad applicability of our approach. And the proposed multi-octave MIR frequency comb is relevant for applications such as dual-comb spectroscopy and trace-gas sensing.

**Graphical Abstract:**

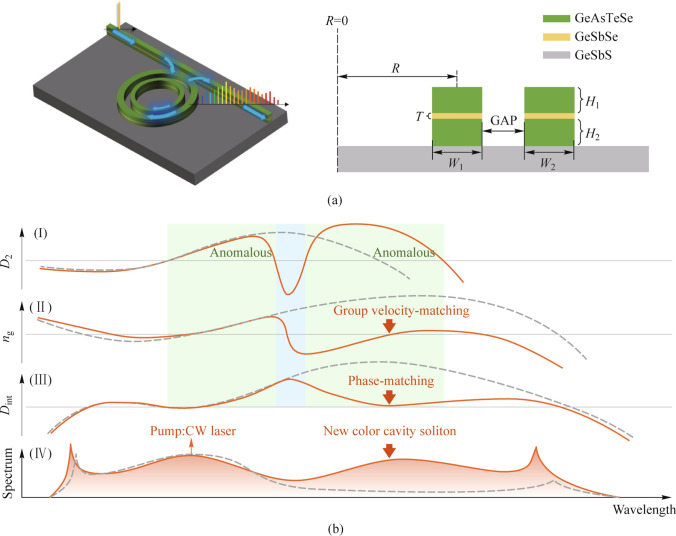

## Introduction

Mid-infrared (MIR) spectral region is critically important as its two atmospheric windows and stronger absorption strength of many molecules than those in the near-infrared (NIR) spectrum [1], which are particularly attractive in applications ranging from materials science to environmental monitoring [2]. Optical frequency combs, which provide equidistant frequency markers in the infrared, visible, and ultraviolet regions, have triggered substantial advances in optical frequency metrology and precision measurements [3, 4]. Over the past decade, advances in microfabrication technology have enabled the development of microresonator-based Kerr frequency combs, which are compact size, broadband, and suitable for building a portable device [5–7]. However, the generation of compact, multi-octave-spanning MIR frequency combs continue to pose significant challenges due to the absence of suitable MIR continuous-wave (CW) pump lasers and appropriate photonic materials for the core and cladding of integrated devices [8, 9].

A promising solution lies in the development of multicolor solitons, which offers broad spectral bandwidths and flexible pump wavelength locations [10–12]. These solitons generate multiple soliton-like components through inter-soliton Cherenkov radiation, each exhibiting a sech^2^ envelope, while behaving as a single pulse in the time domain due to their similar group velocities [13]. Supporting a multicolor soliton state requires multiple separated anomalous dispersion regions, necessitating intricate dispersion engineering [9]. Chalcogenide glasses (ChG), comprising one or more chalcogen elements such as sulfur, selenium, and tellurium (S, Se, Te), along with various metals or non-metals, demonstrate exceptional optical transmission properties that span from the visible to the far-infrared regions (> 25 μm) and possess an amorphous structure conducive to multilayer deposition on silicon wafers via thermal evaporation [14, 15]. Moreover, their optical characteristics can be tuned by manipulating the elemental composition, which is advantageous for achieving flexible dispersion engineering in MIR photonic devices [16]. In recent years, integrated chalcogenide glass photonic devices have attracted significant interest due to their versatile applications in supercontinuum generation, Raman and Brillouin lasers, parametric oscillation, and integrated photonic computing, marking them as pivotal components in the advancement of photonic technologies [17–19]. In addition, the 2-μm band, at the leading position among the new wavelength bands, has developed mature devices, such as commercial 2-μm distributed feedback (DFB) lasers and thulium-doped fiber amplifiers (TDFA) with more than 240 nm gain bandwidth and low noise figure, which is utilized in various application including gas sensing, optical interconnection, and medical treatment [20–23].

In this work, we propose a novel microresonator architecture termed the slot-concentric-dual-ring (SCDR) based on ChGs with low absorption loss and high optical stability in the MIR region [7, 24], including Ge_15_As_20_Te_45_Se_15_ and Ge_15_Sb_15_Se_70_. The SCDR microresonator promotes mode coupling, which enables group velocity matching and the formation of two distinct regions of anomalous dispersion, both essential for supporting a two-color soliton. The introduction of a slot structure within the microresonator allows for precise engineering of the integrated dispersion curve, thereby enabling the tunability for the phase-matching location of new-color and broadening the comb’s bandwidth to span multi-octave. Utilizing a CW pump in the 2-μm band, a multi-octave spanning two-color soliton, extending from 1156.07 to 5054.95 nm (200 THz) has been achieved. Our simulations also demonstrate spectral locking, which stabilizes the generation of frequency combs and offers flexibility in pump wavelength selection. The SCDR microresonator provides a compact and efficient solution for generating broadband MIR frequency combs with customizable spectral profiles, suitable for advanced spectra for medical diagnosis, environmental monitoring and materials science.

## Operation principle

### Principle of two-color soliton generation and microresonator design

**Fig. 1:**
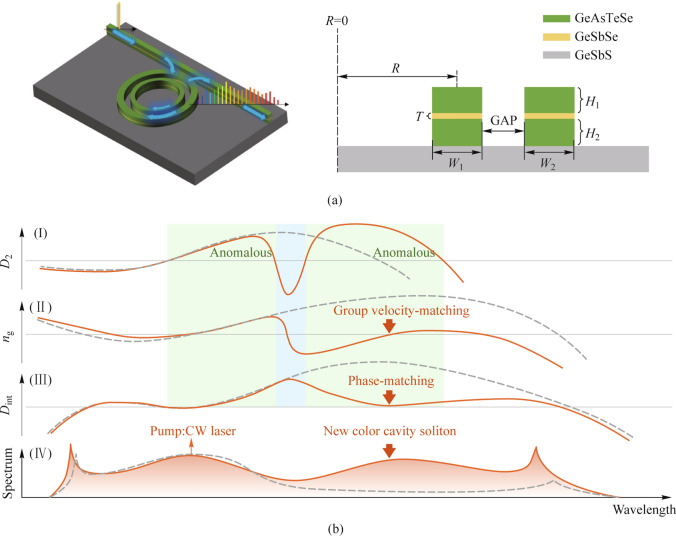
Schematic of the proposed SCDR microresonator and principles for the generation of two-color soliton. **a** 3D profile and cross-section of the SCDR microresonator. **b** Schematic for the generation of broadband MIR two-color soliton based on the SCDR, for comparison a Slot microresonator with the same materials is added, shown by the red curve and gray dashed line, respectively. (I) *D*_2_ profiles of the microresonators. Two separated anomalous dispersion regions and a domain affected by mode coupling of SCDR are colored in green and blue, respectively. (II) *n*_g_ profiles with* x* label indicating value of the pump, provides group velocity-matching (red arrow) for the new-color. (III) *D*_int_ profiles with phase-matching (red arrow) for the new-color. (IV) Spectra for the microresonators with Slot and SCDR structures, respectively. In contrast to the Slot, the SCDR generates a broadband MIR comb with a new-color soliton (red arrow)

Two-color soliton state comprises dual soliton-like components that engage in energy exchange via inter-soliton Cherenkov radiation. These components maintain approximately equal amplitudes while exhibiting distinctly different central frequencies [10]. Such a soliton state is enabled by group-velocity matched co-propagation of two colors in two separated anomalous dispersion regions [25]. In this work, we propose the SCDR microresonator, consisting of concentric inner and outer rings, each featuring a horizontal GeSbSe slot. These are encapsulated by dual GeAsTeSe layers atop a ChG substrate (Ge_25_Sb_10_S_65_, GeSbS), see Fig. 1a. Chalcogenide materials providing broad transparency windows, high refractive indices and nonlinear refractive index coefficients at 2 μm are selected in this work, detailed in Table 1. Mode hybridization arises when the optical path lengths (OPLs) of these concentric rings align, due to coupling between the rings, with the OPLs for each ring being independently calculable [26].

**Table 1:** Properties of integrated ChG materials in this work [27]

Material	*n*	*n*_2_ (10^−17^m^2^/W)	Transparency window (μm)
Ge_15_As_20_Te_45_Se_15_	3.25	3.38	1.5–20
Ge_15_Sb_15_Se_70_	2.64	0.78	1.2–17
Ge_25_Sb_10_S_65_	2.23	0.21	0.5–10

The hybridization couples the modes of the inner and outer rings, resulting in the formation of a pair of symmetric and antisymmetric modes, each exhibiting distinct free spectral range (FSR) behaviors as the wavelength increases. The antisymmetric mode, with its decreasing FSR, induces additional anomalous dispersion in the coupling region, a feature exploited to achieve multiple zero dispersion wavelengths [28]. Conversely, the symmetric mode exhibits an increased FSR, resulting in normal dispersion around the coupling region. To create two separate regions of anomalous dispersion, the symmetric mode is selected, as shown in Fig. 1b (I). Additionally, group-velocity matching for the co-propagation of two colors is required, which can be calculated by

1 $${v}_{{\text{g}}} = {c/n}_{{\text{g}}} ,$$

where *n*_g_ is the group index of modes given by

2 $${n}_{{\text{g}}} { = }\frac{{c}}{{{2}\uppi \cdot {\text{FSR}} \cdot {R}}},$$

where* R* is the radius of the microresonator. The decrease of *n*_g_ induced by the symmetric mode coupling enables the match of *n*_g_ for the two separated anomalous regions, as shown in Fig. 1b (II). Moreover, to facilitate inter-soliton Cherenkov radiation, phase-matching between the pump and the new color is required [9, 10, 13], which can be approximated by

3 $${D}_{{{\text{int}}}} = \omega_{\mu } - \omega_{0} - {D}_{{1}} \mu { = }\mathop \sum \limits_{{k = 2}}^{\infty } \frac{1}{{k{!}}}{D}_{{k}} \mu^{{k}} { } \approx { }0,$$

where $\omega_{\mu }$ are the resonance frequencies of the microresonators, determined by

4 $$\omega_{\mu } = \omega_{0} +{D}_{{1}} \mu + \frac{{1}}{{2}}{D}_{{2}} \mu^{{2}} + \cdots,$$

where *D*_2_ is the second-order dispersion. *D*_int_ represents the integrated dispersion, *D*_*k*_ presents the *k*-order of the dispersion coefficient, $\omega_{0\left( \mu \right)}$ is the angular resonant frequency for pump mode and other modes, and *μ* is the relative mode number. The strong anomalous dispersion, induced by symmetric mode coupling, decrease the *D*_int_ profile so as to garner the phase-matching between the two colors, see Fig. 1b (III). When group velocity and phase for the new-color soliton are matched at the same wavelength, located in another anomalous dispersion, a new-color soliton is generated, which features roughly similar amplitudes but distinctly different center frequencies with the pump, as shown in Fig. 1b (IV).

For comparative purposes, the schematic for the generation of broadband MIR two-color soliton based on a Slot microresonator fabricated from the same materials is depicted in Fig. 1b. This microresonator, unaffected by mode hybridization, exhibits a smoother dispersion profile with a single anomalous dispersion region. The Slot microresonator’s *D*_int_ profile escalates at longer wavelengths due to the accumulation of anomalous dispersion, precluding the support of a two-color soliton. Consequently, it generates only a traditional soliton with a sech^2^ envelope, its bandwidth governed by the *D*_2_ profile [29]. Moreover, the elevated *D*_int_ presents a barrier too high to sustain a high-amplitude dispersive wave (DW) at longer wavelengths [30, 31]. In contrast, the SCDR microresonator facilitates the generation of a new-color soliton, which not only matches the soliton supported by the pump in amplitude but also differs significantly in frequency, fulfilling the criteria for a two-color soliton. This new-color soliton, combined with a DW, is tailored for the MIR band, thereby effectively expanding the bandwidth of the comb and enhancing the energy at longer wavelengths.

**Fig. 2:**
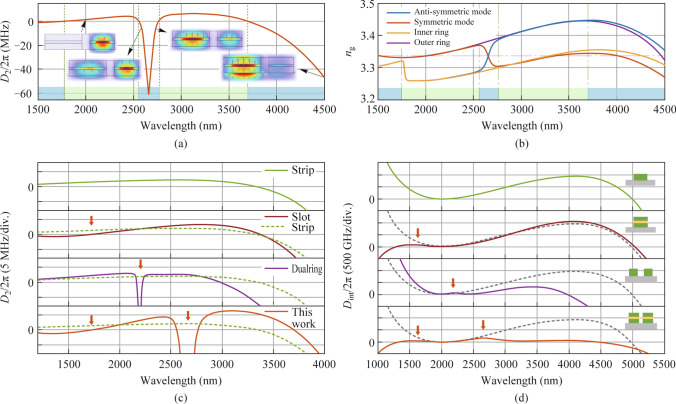
Simulation of modified geometric parameters for SCDR supporting two-color soliton, compared with other structures. **a** Dispersion profile (*D*_2_) and guide mode distribution of the SCDR, with two anomalous dispersion domains (green areas) separated by normal dispersion (blue area). **b** Group indices of the SCDR for symmetric mode (red) and antisymmetric mode (blue), compared with isolated inner ring (yellow) and outer ring (purple) of the SCDR. The pink dashed line shows the group index of the pump. **c** Second-order dispersion profiles of four different microresonators. Detailed parameters: (1) Strip microresonator, *W* = 1.6 μm, *H* = 550 nm, *R* = 200 μm. (2) Slot microresonator, *W* = 1.5 μm, *H*_1_ = 350 nm, *H*_2_ = 400 nm, *t* = 150 nm, *R* = 200 μm. (3) Dualring microresonator, *W*_1_ = 2.4 μm, *W*_2_ = 1.6 μm, *H* = 700 nm, Gap = 500 nm. **d** Integrated dispersion and the cross-section of the four microresonators

We simulate the generation of a broadband MIR comb using a 2 μm pump by engineering the dispersion of the SCDR, see Fig. 2. Only TM modes are considered in this work, which are easier to realize group-velocity and phase matching conditions at the same wavelength. While the group-velocity and phase of TE modes matched far from each other, which is hard to realize two-color soliton. Meanwhile, the guided modes for TM modes are finely confined in the Slot layer, achieving flexible tunability. In general, the guided mode is mainly confined in the high-index Slot layer, resulting in the dispersion profile at short wavelengths similar to the isolate outer-ring [32]. After mode hybridization, it resembles the isolated inner ring’s dispersion profile. Mode coupling is designed to occur around 2700 nm, featuring strong normal dispersion and separating the two anomalous dispersion regions. To verify the affection of the mode coupling, the inner and outer rings of the SCDR are simulated independently. Among the four modes, only the symmetric mode enables group index matching for the new-color soliton at 3360.5 nm, provided by the negative slope of *n*_g_ around 2700 nm due to mode hybridization.

For comparison, we simulate the *D*_2_ and *D*_int_ profiles of traditional microresonators such as Strip, Slot, and Dualring microresonators, adjusted for the MIR region using a 2 μm pump (Fig. 2c, d). The Strip microresonator, with limited design flexibility, cannot maintain a low *D*_int_ barrier for high amplitude DW in the MIR band [30, 31], resulting in a narrowband soliton. The Slot microresonator offers additional design freedom to adjust dispersion via the anti-crossing effect caused by mode transition, extending the comb’s bandwidth through DW [33–36]. However, its spectrum is still based on the sech^2^-shaped *D*_2_ profile, preventing a broadband MIR comb with a 2 μm pump. The Dualring microresonator, enabled by mode hybridization, achieves group velocity and phase-matching for a two-color soliton. However, the introduced normal dispersion confines the new color close to the pump, providing narrow bandwidth [9, 37]. Our SCDR microresonator combines the advantages of Slot and Dualring microresonators, providing tunability and realizable two-color soliton. Consequently, group index and phase-matching of new-color are designed to coincide at 3360.5 and 3360.16 nm, respectively. Thus, a new-color soliton is expected around 3360 nm, with similar amplitudes to the pump-supported soliton. Additionally, the Slot structure’s tunability achieves flat and broad *D*_int_ at longer wavelengths, enhancing the DW amplitude around 4487.64 nm, greatly extending the bandwidth and energy in the MIR region. The DW around 1312.22 nm also broadens the bandwidth.

### Dispersion engineering

To obtain a new-color soliton with a high comb power in the MIR region, the *D*_int_ profile should be finely adjusted, since the phase-matching for new-color and the generation of DWs are approximated by *D*_int_ = 0. Meanwhile, a low *D*_int_ barrier is required for the generation of Cherenkov radiation [37], enabling the two-color soliton and DWs. Therefore, the tunability of our SCDR microresonator is necessary to be verified by simulating the six structural parameters individually. The standard profile (yellow) satisfies the requirements for two-color soliton discussed above, with the same geometric parameters in Fig. 2a.

**Fig. 3:**
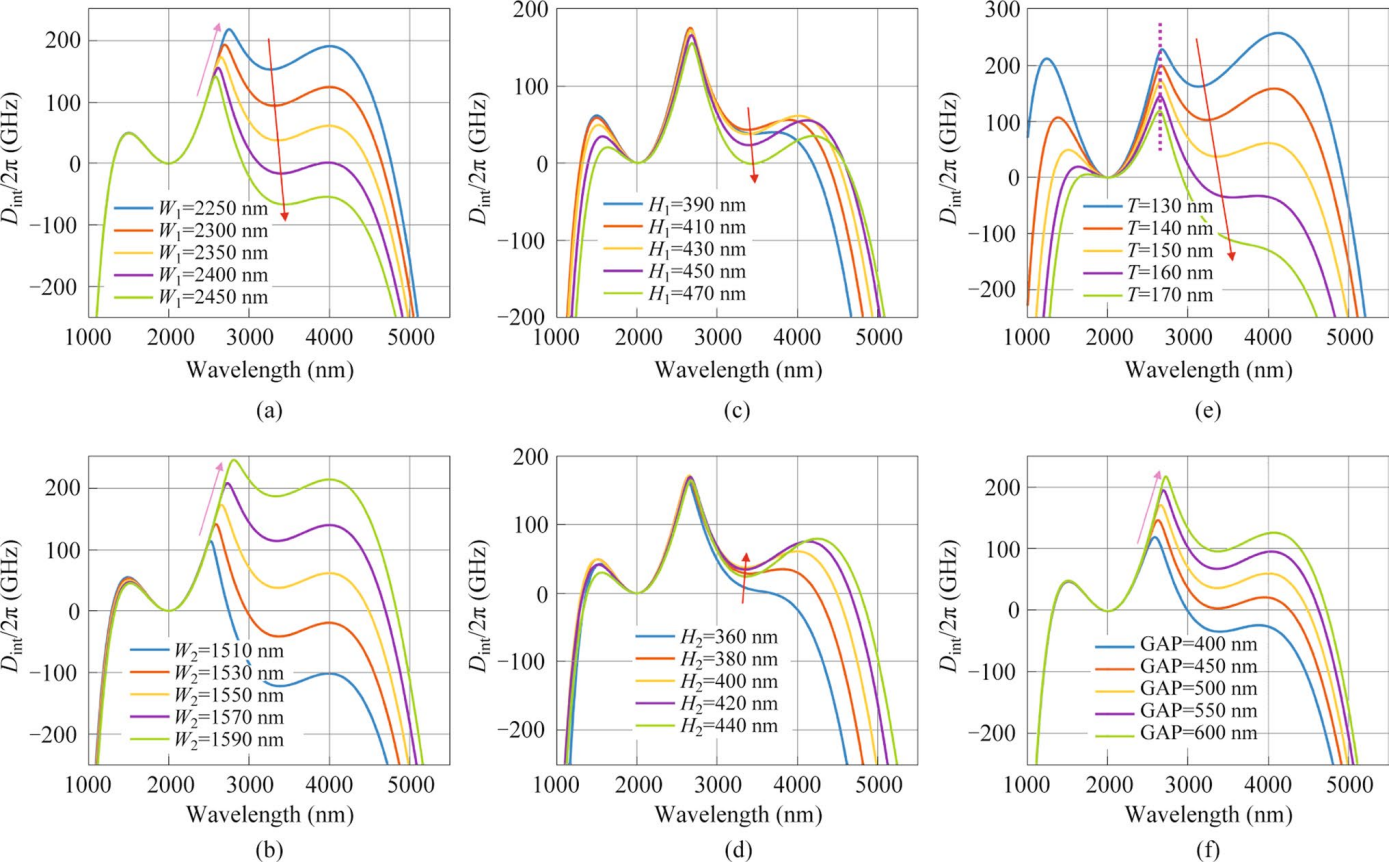
Integrated dispersion profiles with the variation of **a** inner waveguide width *W*_1_, **b** outer waveguide width *W*_2_, **c** upper strip waveguide height *H*_1_, **d** lower strip waveguide height *H*_2_, **e** slot layer thickness *T*, and **f** interval between the concentric rings gap

As shown in Fig. 3b and f, increasing *W*_2_ or the gap gradually shifts the *D*_int_ profile at longer wavelengths from negative to positive, allowing adjustment of the phase mismatch for the new-color without changing its position. While increasing *W*_1_, *H*_1_, *H*_2_, and *T* regulate the phase-matching wavelength for the new-color to longer wavelength, as indicated by the red arrows in Fig. 3a, c, d, and e. The *D*_int_ barrier caused by mode coupling can be effectively reduced by increasing *W*_1_ or *T*, or by decreasing *W*_2_ and the gap, as illustrated in Fig. 3a, b, e, and f. Moreover, the DW before mode coupling can be shifted to shorter wavelengths, further broadening the comb’s bandwidth, by decreasing *H*_1_ and *T*, as seen in Fig. 3c and e. Conversely, the *D*_int_ profile, which determines the phase-matching location and barrier for Cherenkov radiation at longer wavelengths, can be independently tailored while preserving the profile at shorter wavelengths, as shown in Fig. 3a and f. In addition, as variation of *T* affects the *D*_int_ profile a lot, thickness of the Slot layer requires be precisely controlled with a resolution down to the few nanometers level, as seen in Fig. 3e. By adjusting these structural parameters, the locations of the phase-matching and the height of the *D*_int_ barrier can be precisely tuned.

## Results and discussion

### Two-color soliton generation

**Fig. 4:**
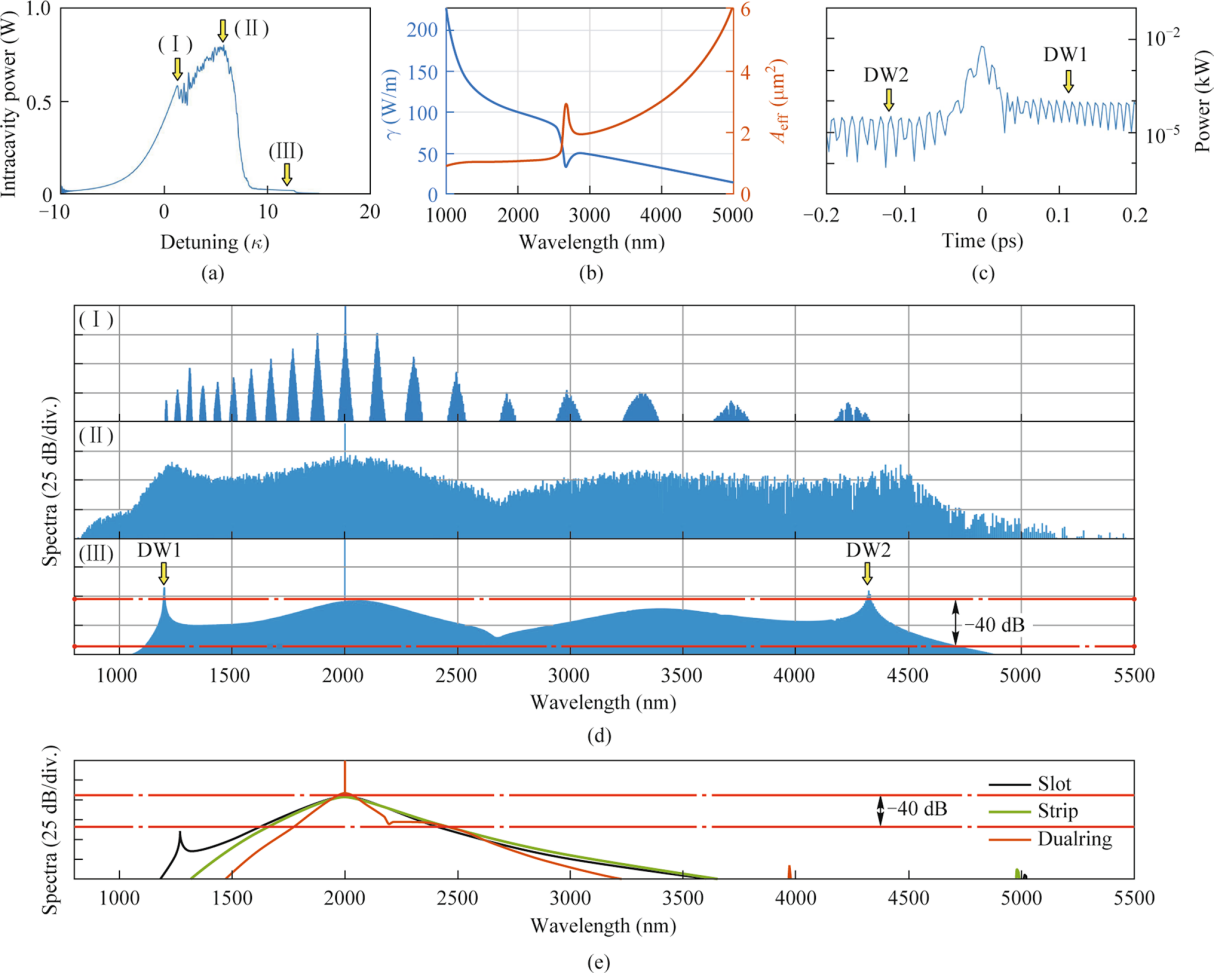
Generation of the two-color solitons. **a** Intracavity power changes with the pump resonance scanning. **b** Nonlinear coefficient (blue) and effective mode area (red) of the SCDR versus wavelengths. **c** Temporal profile of the two-color soliton state. Temporal profile has a modulated envelope due to the beating between the two-color regions. **d** Spectral profiles of two-color soliton at positions corresponding to **a**. **e** Spectral profiles of the soliton state of the conventional structures (Dualring, red; Slot, black; Strip, green)

To study the generation of a two-color soliton comb, we simulate two-color soliton using the Lugiato-Lefever equation in the integrated chalcogenide SCDR microresonator [38],

5 $$\frac{{\partial \widetilde{A}_{\mu } \left( {t} \right)}}{{\partial {t}}}{ = }\left( { - \frac{k}{{2}}{\text{ + i}}\left( {{2}\uppi \delta_{0} } \right){\text{ + i}}D_{{{\text{int}}}} \left( \mu \right)} \right)\,\widetilde{A}_{\mu } - {\text{i}}g\left( \mu \right){F}\left[ {\left| {A} \right|^{{2}} {A}} \right]_{\mu } { + }\sqrt {k_{{\text{c}}} } {S}_{{{\text{in}}}} ,$$

where $$A$$ represents the temporal envelopes of the optical field in the microresonators. $${\tilde{A}}_{\mu } {{ = F[A(t)]}}$$, the Fourier transform of the optical field in the azimuthal direction, $$\mu$$ is an integer representing the relative mode number from the pumped wavelength, $$k$$ is the cavity total decay rate $$k = k_{{\text{i}}} + k_{{\text{c}}} = \frac{{\omega }}{{{Q}_{{\text{i}}} }} + \frac{{\omega }}{{{Q}_{{\text{c}}} }}$$ composed of the intrinsic decay rate $$k_\text{i}$$ and the external coupling rate $$k_\text{c}$$.We set $${Q}_{\text{i}}$$ is equal to $${Q}_{\text{c}}$$ to obtain the critical coupling of the microresonator and $${Q}_{\text{c}}$$ is set as $$1\times {10}^{6}$$ at 2 μm. $$\delta_{0}$$ is the pump resonance detuning. $${g = }\frac{{{h}\omega^{{2}} {cn}_{{2}} }}{{{n}_{{\text{g}}}^{{2}} {V}_{{{\text{eff}}}} { }}}$$ represents the Kerr gain coefficient. $${P}_{\text{in}} = {\text{|}{S}_{\text{in}}\text{|}}^{2} \, \text{=} \, \text{60 mW}$$ is the input pump power and $${D}_{\text{int}}$$$$\left( \mu \right)$$ means the integrated dispersion. Considering that the mode area *A*_eff_ will have an in-negligible variation in the MIR region and here to accurately model two-color cavity soliton in the MIR region, the nonlinear reduction induced by mode area (shown in Fig. 4b), high-order dispersion, and self-steepening are included in our model. The mode area *A*_eff_ and nonlinear coefficient *γ* can be calculated by

6 $$A_{{{\text{eff}}}} = \frac{{\left( {\iint {\left| {E\left( {x,y} \right)} \right|^{2} {{\text{d}x\text{d}y}}}} \right)}}{{\left( {\iint {_{\text{core}} \left| {E\left( {x,y} \right)} \right|^{4} {{\text{d}x\text{d}y}}}} \right)}},$$

7 $${{\gamma}} = \frac{{{n}_{{2}} \omega }}{{cA_{{{\text{eff}}}} }},$$

where $E\left( {x,y} \right)$ is the profile of the field, ${n}_{{2}} = 3.4 \times {10}^{{ - 17}} {\text{ m}}^{{2}} {\text{/W}}$ is the nonlinear Kerr index for GeSbSe at 2 μm. As shown in Fig. 4b, the nonlinear coefficient *γ* is inversely proportional to the mode area *A*_eff_. Due to the mode coupling process, there is a bump in the *A*_eff_ curve, causing a heavy reduction of the nonlinear coefficient *γ*.

We begin by theoretically studying the dynamic of two-color soliton with the pump wavelength of 2 μm and the pump power of 60 mW, see Fig. 4. By uniformly tuning pump resonance detuning from – 10 *κ* to 15 *κ*, we observed the variation of intracavity power in the SCDR microresonator (Fig. 4a). As the pump detuning shifts from blue detuned to 1.41 *κ* (I), 5.73 *κ* (II), and 12.44 *κ* (III), the system transitions through sub-combs, modulation instability, and a phase-locked two-color soliton state, respectively. Initially, sub-combs form around the primary lines in the blue-detuned region (Fig. 4d (I)). As the pump wavelength redshifts, the sub-combs merge, leading to a chaotic modulation instability (Fig. 4d (II)). Further redshift reaches the red-detuned side, where the resonance exhibits bistable behavior, resulting in a phase-locked two-color soliton state (Fig. 4d (III)). Interestingly, additional anomalous dispersion in the MIR region can facilitate the exchange of energy between two-color soliton through Cherenkov radiation [13]. Hence, a broadband two-color soliton frequency comb can be generated, spanning from 1098.39 to 4770.35 nm at − 40 dB level, and the spectral profile shows two characteristic sech^2^ envelopes at the two phase-matched regions. Meanwhile, two DWs are stimulated at 1202.36 and 4320.72 nm, meeting the satisfaction of the phase matching condition. The spectrum in the two-color regions shows a beating component, creating a modulated envelope for the temporal pulse of the two-color soliton (Fig. 4c) [9]. Furthermore, the two-color soliton temporal pulse is beneath the two backgrounds, further proving the emission of two DWs [39].

For comparison, we also numerically investigate the generation of the soliton comb state in the Slot, Strip, and Dualring microresonator with a pump power of 60 mW. As shown in Fig. 4e, the Strip microresonator, despite achieving phase matching in the MIR region, only produces a single sech^2^ envelope at the pump wavelength due to a high *D*_int_ barrier, failing to extend into the MIR region (see Fig. 2). The Slot microresonator, while offering more design flexibility, can achieve another DW at 1273.39 nm but is still unable to flatten the *D*_int_ barrier in the MIR region. For the Dualring resonator, additional anomalous dispersion can also be achieved around 2.3 μm, resulting in phase-matching that allows Cherenkov radiation. However, the presence of a high *D*_int_ barrier in the MIR region limits its bandwidth expansion to the MIR region. By integrating the dispersion characteristics of the Slot and Dualring, the SCDR microresonator not only achieves a broadband, close-to-zero dispersion in the MIR region. This design supports a significantly broader MIR frequency comb, enabling coverage of MIR wavelengths that conventional structures cannot achieve.

### Pump wavelength selectivity

**Fig. 5:**
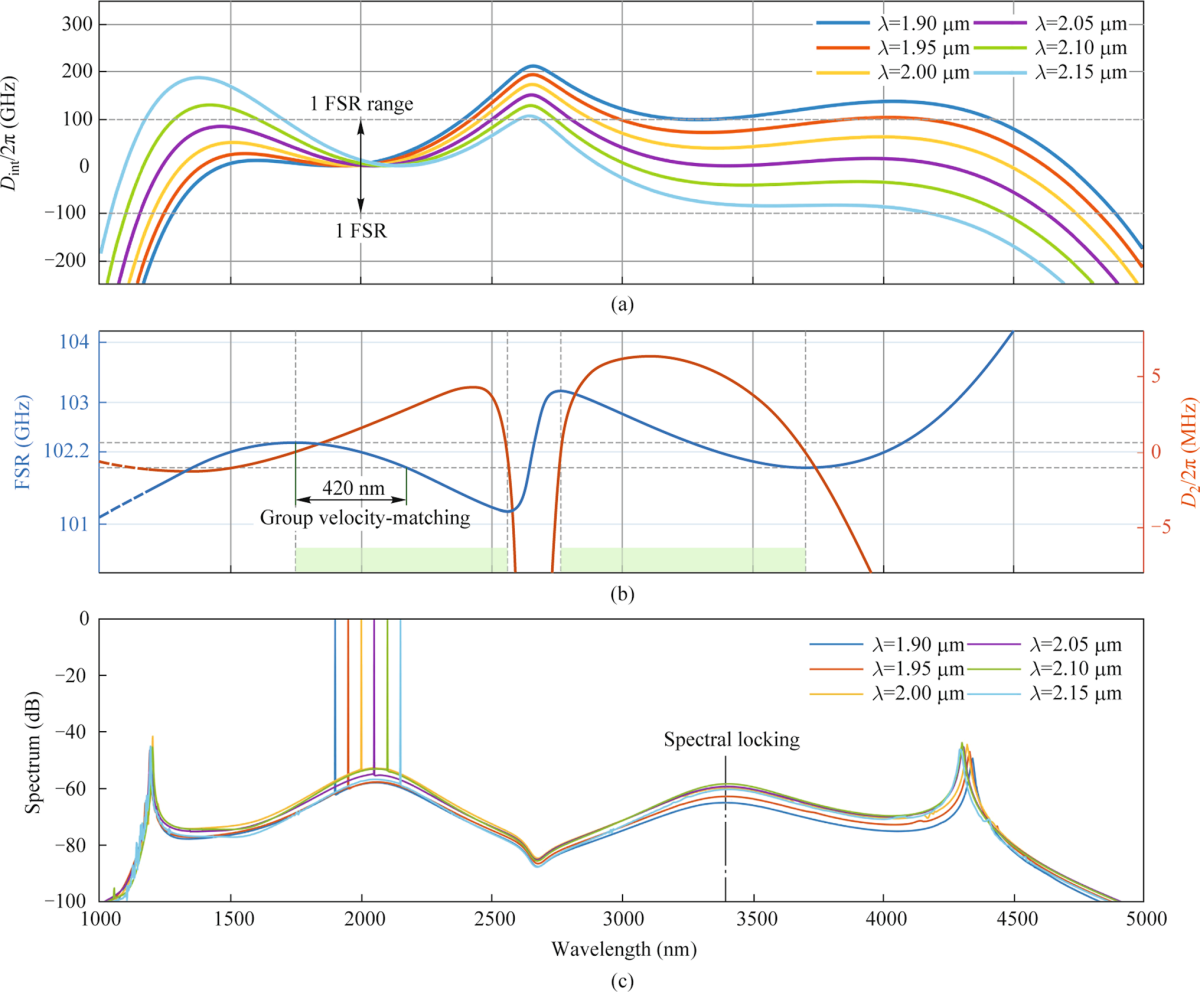
Limitations and comb spectra of two-color cavity soliton at different pump wavelengths. **a** Allowed *D*_int_ barrier is 1 FSR. **b** Tunable range for the pump is 420 nm, limited by the group velocity-matching. **c** Comb spectra corresponding to the various pump wavelengths. Spectral locking characteristic is found during simulation

We concentrate on a pump wavelength around 2 μm, supported by readily available commercial distributed DFB feedback lasers and TDFA. Considering the effect of the *D*_int_ barrier, here we select the *D*_int_ barrier within one FSR region in different pump wavelengths to effectively generate Cherenkov radiation, which corresponds to the pump wavelength (*λ*_p_) adjusting from 1.90 to 2.15 μm, see Fig. 5a. Meanwhile, the requirement for group velocity-matching between the pump and the new-color soliton, which must reside in the anomalous dispersion regions, restricts the *λ*_p_ flexibility to a range from 1748.42 to 2173.37 nm, see Fig. 5b. When these conditions are both satisfied, inter-soliton Cherenkov radiation is facilitated within the spectral region, resulting in the generation of a two-color soliton [13]. To ensure a precise comparison of the two-color soliton in different *λ*_p_, we employ nearly identical pump powers in the aforementioned above LLE model.

As *λ*_p_ is equal to 1.90 μm, *D*_int_ barrier in the MIR region is close to about one FSR, enabling effective Cherenkov radiation and energy exchange with this MIR color, leading to a two-color soliton with a bandwidth covering the MIR region. As *λ*_p_ redshifts from 1.90 to 2.05 μm, the *D*_int_ barrier in the MIR region can be reduced to about zero and the comb power of the MIR color raises, respectively. Beyond 2.05 μm, the *D*_int_ barrier in the MIR region boosts again, causing a slight reduction of the comb power. Furthermore, as a new color is generated, the spectral profiles of the two-color soliton exhibit spectral locking characteristics, meaning that the spectral profiles of soliton can be sustained even when the *λ*_p_ fluctuates in a large range (Fig. 5c). This stability is attributed to strong inter-soliton nonlinear interactions where phase and group velocity matching are optimally satisfied [9]. Therefore, the *λ*_p_ selectivity for generating two-color soliton can be robust, enabling the continued generation of the MIR color in the inner ring. Moreover, by combining a commercial DFB laser with thulium-doped fiber amplifiers, the selection of *λ*_p_ for generating two-color solitons can be flexibly managed with a pump power of approximately 60 mW.

### On-demand tunable MIR frequency comb

**Fig. 6:**
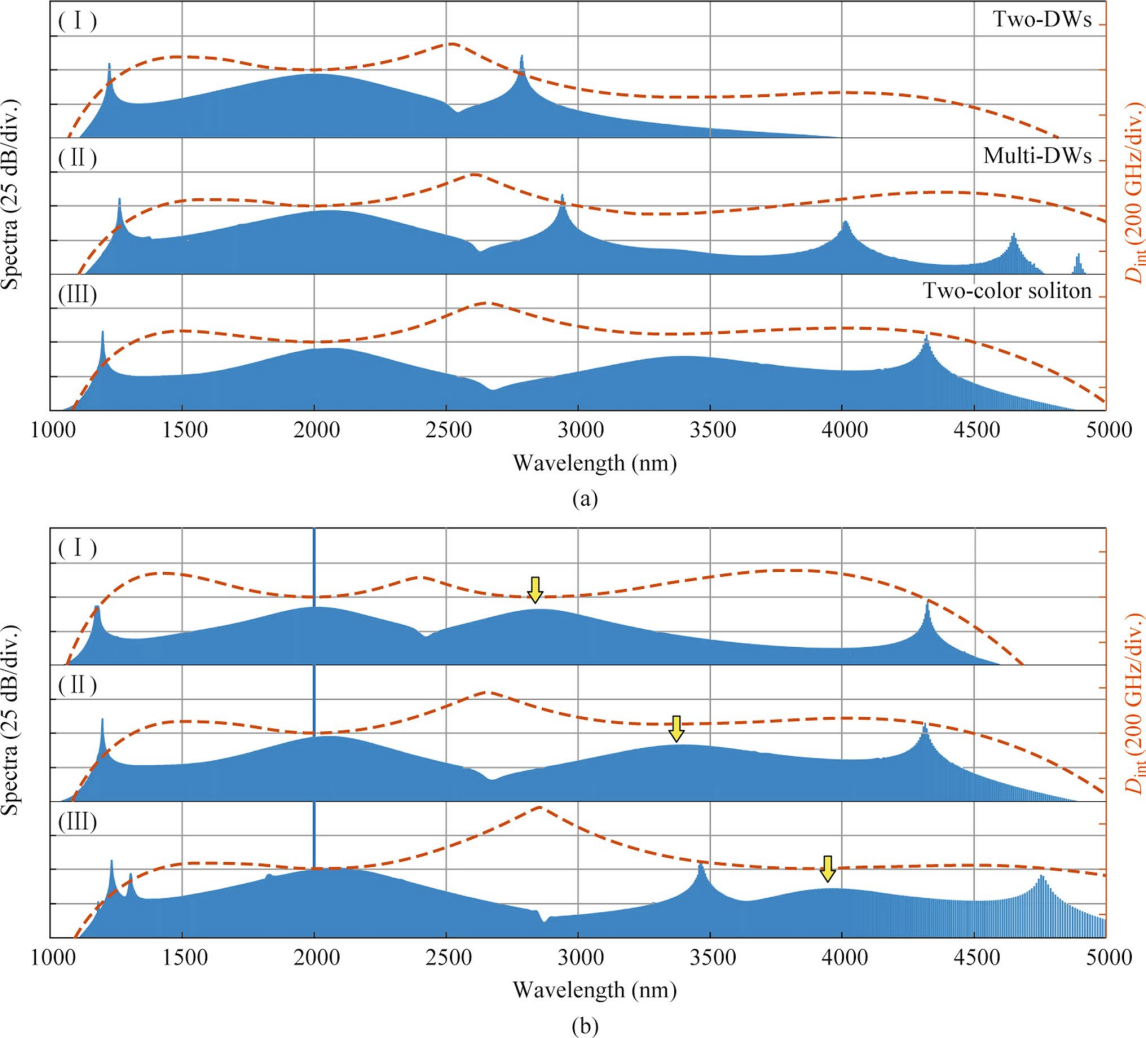
Integration dispersion (right axis) and comb spectra (left axis) of the microresonators corresponding to the geometric parameters in Table 2. **a** Comparison between the emission of DWs and a new color. **b** Spectral profiles correspond to the different positions of the MIR color

In this section, we have investigated the influence of the geometries to the spectral profiles of the two-color soliton. However, by tailoring the geometries of the SCDR microresonator, the group velocity condition for generating a new-color can possibly be broken. As shown in Fig. 6a (I), two-DWs are generated without a new-color in the MIR region due to the break of the group velocity matching. Furthermore, the *D*_int_ in Fig. 6a (II) seems to support a new-color while its group velocity is not in the phase-matched region. Therefore, multi-DWs state is more competitive than the new-color state during the nonlinear process. It is noteworthy that a key dispersion characteristic of the SCDR microresonator allows for the independent tailoring of *D*_int_ in the MIR band, while preserving the *D*_int_ profiles in the short wavelength region. Thereby, under the conditions required for generating a new-color soliton, the phase-matched region in the MIR region can also be adjusted without affecting the *D*_int_ profiles of the short wavelength, which provides a convenient approach to expanding the bandwidth of the two-color soliton to the MIR region. By tailoring the geometries of the SCDR microresonator, the position of the new-color soliton can be varied from 2849.76 nm in (I) to 3399.49 nm in (II), without altering the spectrum at the short wavelength. Ultimately, a multi-octave MIR frequency comb spanning from 1156.07 to 5054.95 nm can be obtained by further modifying the position of the MIR color to longer wavelengths, see Fig. 6b (III).

**Table 2:** Geometric parameters of the SCDR microresonators correspond to Fig. 6

Structure	*W*_1_ (nm)	*W*_2_ (nm)	*H*_1_ (nm)	*H*_2_ (nm)	*T* (nm)	GAP (nm)	*R* (μm)
(a) I	2350	1510	430	400	150	500	140
(a) II	2350	1550	430	450	150	550	150
(a) III	2350	1550	430	400	150	500	140
(b) I	2100	1550	420	340	140	510	200
(b) II	2350	1550	430	400	150	500	140
(b) III	2600	1520	480	450	150	800	120

### Schematic of fabrication procedures

**Fig. 7:**
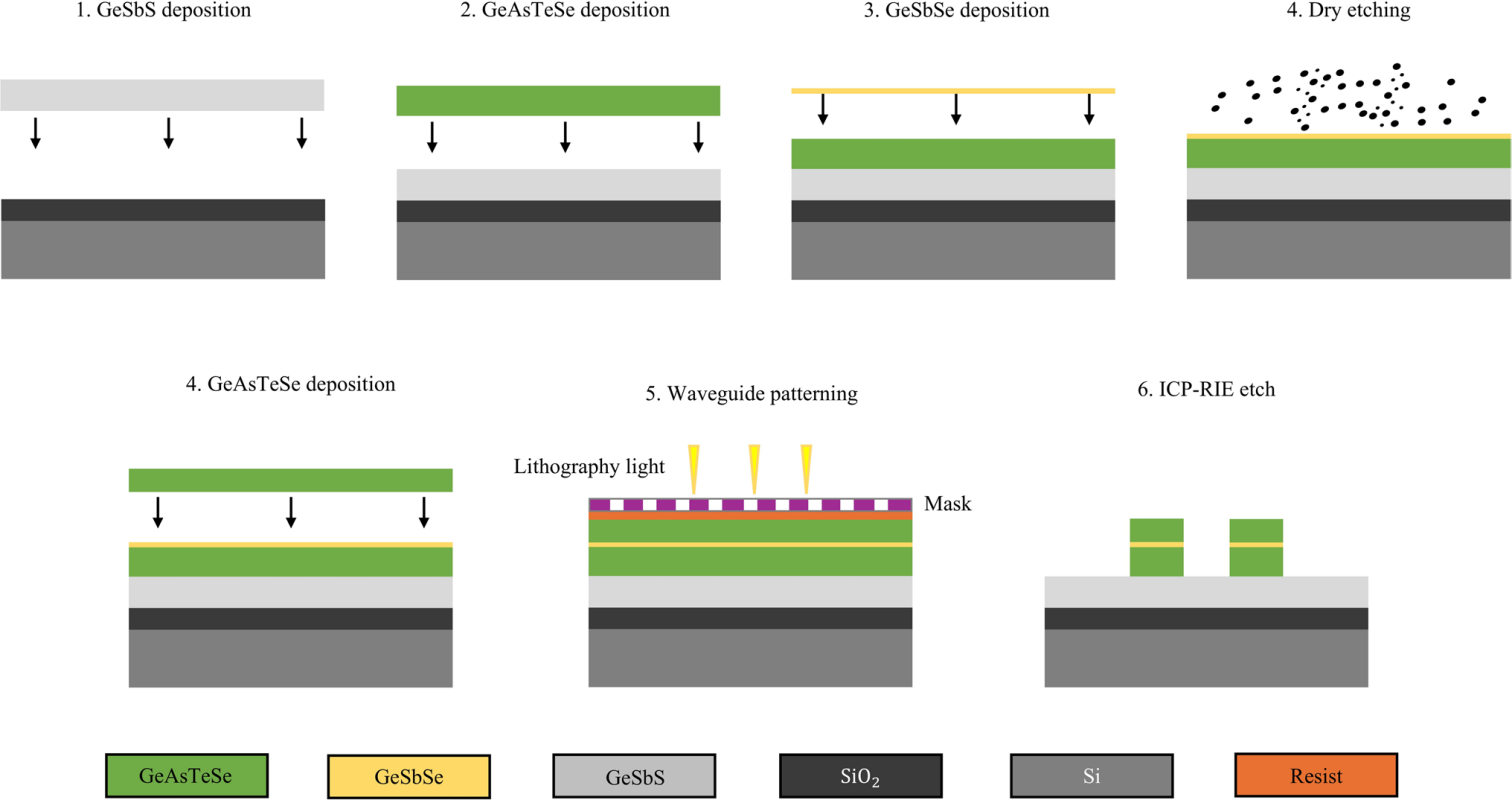
Schematic of the fabrication procedures for the SCDR microresonator

A feasible nano-fabrication procedure for our SCDR microresonator is demonstrated in Fig. 7. First, a 5 μm thick GeSbS lower layer is deposited by thermal evaporation as the substrate of the SCDR on a silicon with a 3 μm thick thermal oxidation layer to avoid the severe absorption of silica in MIR region. Then, a 0.4 μm thick GeAsTeSe lower layer and a 0.15 μm thick GeSbSe slot layer are deposited successively by thermal evaporation. Whereafter, a dry etch trimming approach is utilized to fine control of the thickness of the slot layer with a resolution down to the few nanometers level [29]. Subsequently, another 0.43 μm thick GeAsTeSe layer is deposited by thermal evaporation as the upper layer of the core. After film deposition, the photoresist is spin-coated onto the wafer, and the waveguide pattern is transferred using a mask. Afterwards, ultraviolet lithography and inductively coupled plasma etching (ICP-RIE) are used to pattern the waveguide, and then the residual photoresist of the chip is removed. The upper layers and gap are etched in an ICP reactive ion etcher with CHF_3_ gas. Moreover, a 3 nm thick Al_2_O_3_ layer on the top surface of the ChG film was deposited to prevent the ChG waveguide’ top surface from oxidizing during the resist removing process. In final, our SCDR microresonators have been fabricated.

## Conclusion

We compare the spectral bandwidth of the two-color soliton comb in this work with several reported frequency combs in the microresonator, as detailed in Table 3. Considering the bandwidth covering the MIR region, both experimental and numerical works relevant to the MIR are included [5–7, 28, 36, 37, 40, 41]. To the best of our knowledge, the state-of-the-art bandwidth of MIR frequency comb in numerical simulation is about 108 THz, with a spanning from 2.2 to 10.5 μm, which employed the generation of two-DWs. However, a MIR laser with a *λ*_p_ of 4.51 μm that combines both a narrow linewidth and high CW output power is hard to achieve. In addition to using DW generation to expand frequency comb bandwidth, the creation of multi-color solitons can also extend the frequency comb to a multi-octave span based on broadband inter-soliton Cherenkov radiation [13, 30]. However, the broadband frequency comb of [13, 30] do not cover the MIR region. Thus, to achieve a multi-octave-spanning MIR frequency comb, we combine a two-color soliton with two DWs in this work, ultimately achieving coverage of the MIR region with a *λ*_p_ of 2 μm. Our resulting bandwidth of the two-color soliton is comparable to those achieved in the visible and near-infrared regions [30, 31, 33–35, 42].

**Table 3:** Comparison of MIR frequency combs in recent works

Pump wavelength (μm)	Commercialized CW laser	Spectral range (nm)	Reach MIR	Bandwidth (THz)	References
1.0707	✓	678–1897	✗	284	[30]
1.3	✓	1056–1703	✗	108	[31]
1.55	✓	1180–3400	✓	166	[42]
1.55	✓	1106–2082	✗	127	[33]
1.55	✓	1200–2050	✗	104	[34]
1.55	✓	1309–2172	✗	91	[35]
1.6	✓	740–2110	✗	263	[13]
1.75	✓	1406–2671	✓	101	[28]
2.6	✓	2380–2900	✓	23	[36]
3.07	✓	2600–4060	✓	41	[40]
3.5	✗	3040–3920	✓	22	[6]
4.51	✗	2200–10500	✓	108	[5]
4.78	✗	4100–5700	✓	20	[41]
9	✗	6800–12950	✓	21	[7]
**2**	**✓**	**1156**–**5055**	**✓**	**200**	**This work**

In summary, we have developed a novel dispersion-engineered SCDR microresonator that generates a two-color MIR frequency comb. By integrating the dispersion characteristics of Slot and Dualring structures, this microresonator achieves group velocity and phase matching, crucial for inter-soliton Cherenkov radiation and supporting dual-color soliton generation. Utilizing a 2 μm pump, we achieved a multi-octave-spanning spectrum ranging from 1156.07 to 5054.95 nm with a modest pump power of 60 mW. Our analysis of both spectral and temporal profiles reveals a spectral locking feature that enhances pump wavelength selectivity, crucial for stable two-color soliton generation. The SCDR microresonator not only facilitates the generation of multi-octave-spanning, tunable two-color solitons with readily accessible pump sources but also represents a significant advancement in designing tailored broadband MIR frequency combs, which pave the way for a range of applications, including multi-heterodyne [43] and dual-comb spectroscopy [44, 45] of characteristic molecular vibrational transitions and trace-gas sensing within the Earth’s atmospheric transparency window [2].

## Data Availability

The data that support the results of this work are available from the corresponding author, upon reasonable request.

## References

[1] Picqué, N., Hänsch, T.W.: Frequency comb spectroscopy. Nat. Photonics **13**(3), 146–157 (2019)

[2] Schliesser, A., Picqué, N., Hänsch, T.W.: Mid-infrared frequency combs. Nat. Photonics **6**(7), 440–449 (2012)

[3] Kippenberg, T.J., Holzwarth, R., Diddams, S.A.: Microresonator-based optical frequency combs. Science **332**(6029), 555–559 (2011) 21527707 10.1126/science.1193968

[4] Gaeta, A.L., Lipson, M., Kippenberg, T.J.: Photonic-chip-based frequency combs. Nat. Photonics **13**(3), 158–169 (2019)

[5] Guo, Y., Wang, J., Han, Z., Wada, K., Kimerling, L.C., Agarwal, A.M., Michel, J., Zheng, Z., Li, G., Zhang, L.: Power-efficient generation of two-octave mid-IR frequency combs in a germanium microresonator. Nanophotonics **7**(8), 1461–1467 (2018)

[6] Anashkina, E.A., Marisova, M.P., Sorokin, A.A., Andrianov, A.V.: Numerical simulation of mid-infrared optical frequency comb generation in chalcogenide As2S3 microbubble resonators. Photonics **6**(2), 55 (2019)

[7] Lu, S., Lin, G., Xia, D., Wang, Z., Luo, L., Li, Z., Zhang, B.: Broadband mid-infrared frequency comb in integrated chalcogenide microresonator. Photonics **10**(6), 628 (2023)

[8] Lin, H., Luo, Z., Gu, T., Kimerling, L.C., Wada, K., Agarwal, A., Hu, J.: Mid-infrared integrated photonics on silicon: a perspective. Nanophotonics **7**(2), 393–420 (2017)

[9] Moille, G., Li, Q., Kim, S., Westly, D., Srinivasan, K.: Phased-locked two-color single soliton microcombs in dispersion-engineered Si3N4 resonators. Opt. Lett. **43**(12), 2772–2775 (2018) 29905685 10.1364/OL.43.002772 PMC6098686

[10] Melchert, O., Willms, S., Morgner, U., Babushkin, I., Demircan, A.: Crossover from two-frequency pulse compounds to escaping solitons. Sci. Rep. **11**(1), 11190 (2021) 34045603 10.1038/s41598-021-90705-6 PMC8160248

[11] Melchert, O., Willms, S., Bose, S., Yulin, A., Roth, B., Mitschke, F., Morgner, U., Babushkin, I., Demircan, A.: Soliton molecules with two frequencies. Phys. Rev. Lett. **123**(24), 243905 (2019) 31922846 10.1103/PhysRevLett.123.243905

[12] Lourdesamy, J.P., Runge, A.F.J., Alexander, T.J., Hudson, D.D., Blanco-Redondo, A., de Sterke, C.M.: Spectrally periodic pulses for enhancement of optical nonlinear effects. Nat. Phys. **18**(1), 59–66 (2022)

[13] Luo, R., Liang, H., Lin, Q.: Multicolor cavity soliton. Opt. Express **24**(15), 16777–16787 (2016) 27464131 10.1364/OE.24.016777

[14] Eggleton, B.J., Luther-Davies, B., Richardson, K.: Chalcogenide photonics. Nat. Photonics **5**(3), 141–148 (2011)

[15] Petersen, C.R., Møller, U., Kubat, I., Zhou, B., Dupont, S., Ramsay, J., Benson, T., Sujecki, S., Abdel-Moneim, N., Tang, Z., Furniss, D., Seddon, A., Bang, O.: Mid-infrared supercontinuum covering the 1.4–13.3 μm molecular fingerprint region using ultra-high NA chalcogenide step-index fibre. Nat. Photonics **8**(11), 830–834 (2014)

[16] Kim, D.G., Han, S., Hwang, J., Do, I.H., Jeong, D., Lim, J.H., Lee, Y.H., Choi, M., Lee, Y.H., Choi, D.Y., Lee, H.: Universal light-guiding geometry for on-chip resonators having extremely high Q-factor. Nat. Commun.Commun. **11**(1), 5933 (2020) 10.1038/s41467-020-19799-2 PMC7683556 33230207

[17] Xia, D., Huang, Y., Zhang, B., Zeng, P., Zhao, J., Yang, Z., Sun, S., Luo, L., Hu, G., Liu, D., Wang, Z., Li, Y., Guo, H., Li, Z.: Engineered Raman lasing in photonic integrated chalcogenide microresonators. Laser Photonics Rev. **16**(4), 2100443 (2022)

[18] Xia, D., Yang, Z., Zeng, P., Zhang, B., Wu, J., Wang, Z., Zhao, J., Huang, J., Luo, L., Liu, D., Yang, S., Guo, H., Li, Z.: Integrated chalcogenide photonics for microresonator soliton combs. Laser Photonics Rev. **17**(3), 2200219 (2023)

[19] Xia, D., Zhao, J., Cheng, H., Wang, Z., Huang, J., Luo, L., Liu, D., Yang, S., Zhang, B., Li, Z.: Energy dissipation engineering for widely tunable (1.2–2.1 µm) optical parametric oscillation in integrated chalcogenide microresonators. Laser Photonics Rev. (2024)

[20] Shen, W., Zeng, P., Yang, Z., Xia, D., Du, J., Zhang, B., Xu, K., He, Z., Li, Z.: Chalcogenide glass photonic integration for improved 2 μm optical interconnection. Photon. Res. **8**(9), 1484–1490 (2020)

[21] Li, J., Liu, Y., Meng, Y., Xu, K., Du, J., Wang, F., He, Z., Song, Q.: 2 μm wavelength grating coupler, bent waveguide, and tunable microring on silicon photonic MPW. IEEE Photonics Technol. Lett. **30**(5), 471–474 (2018)

[22] Yu, Y., Gai, X., Ma, P., Vu, K., Yang, Z., Wang, R., Choi, D.Y., Madden, S., Luther-Davies, B.: Experimental demonstration of linearly polarized 2–10 μm supercontinuum generation in a chalcogenide rib waveguide. Opt. Lett. **41**(5), 958–961 (2016) 26974090 10.1364/OL.41.000958

[23] Kong, D., Liu, Y., Ren, Z., Jung, Y., Kim, C., Chen, Y., Wheeler, N.V., Petrovich, M.N., Pu, M., Yvind, K., Galili, M., Oxenløwe, L.K., Richardson, D.J., Hu, H.: Super-broadband on-chip continuous spectral translation unlocking coherent optical communications beyond conventional telecom bands. Nat. Commun.Commun. **13**(1), 4139 (2022) 10.1038/s41467-022-31884-2 PMC9288461 35842421

[24] Xia, D., Huang, Y., Zhang, B., Yang, Z., Zeng, P., Shang, H., Cheng, H., Liu, L., Zhang, M., Zhu, Y., Li, Z.: On-chip broadband mid-infrared supercontinuum generation based on highly nonlinear chalcogenide glass waveguides. Front. Phys. **9**, 598091 (2021)

[25] Oreshnikov, I., Melchert, O., Willms, S., Bose, S., Babushkin, I., Demircan, A., Morgner, U., Yulin, A.: Cherenkov radiation and scattering of external dispersive waves by two-color solitons. Phys. Rev. A **106**(5), 053514 (2022)

[26] Kim, S., Han, K., Wang, C., Jaramillo-Villegas, J.A., Xue, X., Bao, C., Xuan, Y., Leaird, D.E., Weiner, A.M., Qi, M.: Dispersion engineering and frequency comb generation in thin silicon nitride concentric microresonators. Nat. Commun.Commun. **8**(1), 372 (2017) 10.1038/s41467-017-00491-x PMC5575100 28851874

[27] Pan, J., Xia, D., Wang, Z., Zhang, B., Li, Z.: Chalcogenide chip-based frequency combs for advanced laser spectroscopy. J. Lightwave Technol. **41**(13), 4065–4078 (2023)

[28] Wang, Z., Luo, L., Xia, D., Lu, S., Lin, G., Gao, S., Li, Z., Zhang, B.: Engineered octave frequency comb in integrated chalcogenide dual-ring microresonators. Front. Photon. **4**, 1066993 (2023)

[29] Moille, G., Westly, D., Orji, N.G., Srinivasan, K.: Tailoring broadband Kerr soliton microcombs via post-fabrication tuning of the geometric dispersion. Appl. Phys. Lett. **119**(12), 121103 (2021) 10.1063/5.0061238 PMC10941269 38496785

[30] Moille, G., Lu, X., Stone, J., Westly, D., Srinivasan, K.: Fourier synthesis dispersion engineering of photonic crystal microrings for broadband frequency combs. Commun. Phys.. Phys. **6**(1), 144 (2023) 10.1038/s42005-023-01253-6 PMC10916593 38450291

[31] Pfeiffer, M.H.P., Herkommer, C., Liu, J., Guo, H., Karpov, M., Lucas, E., Zervas, M., Kippenberg, T.J.: Octave-spanning dissipative Kerr soliton frequency combs in Si3N4 microresonators. Optica **4**(7), 684–691 (2017)

[32] Guo, Y., Jafari, Z., Xu, L., Bao, C., Liao, P., Li, G., Agarwal, A.M., Kimerling, L.C., Michel, J., Willner, A.E., Zhang, L.: Ultra-flat dispersion in an integrated waveguide with five and six zero-dispersion wavelengths for mid-infrared photonics. Photon. Res. **7**(11), 1279–1286 (2019)

[33] Weng, H., Liu, J., Afridi, A.A., Li, J., Dai, J., Ma, X., Zhang, Y., Lu, Q., Donegan, J.F., Guo, W.: Directly accessing octave-spanning dissipative Kerr soliton frequency combs in an AlN microresonator. Photon. Res. **9**(7), 1351–1357 (2021)

[34] Gu, J., Li, X., Qi, K., Pu, K., Li, Z., Zhang, F., Li, T., Xie, Z., Xiao, M., Jiang, X.: Octave-spanning soliton microcomb in silica microdisk resonators. Opt. Lett. **48**(5), 1100–1103 (2023) 36857223 10.1364/OL.479251

[35] Song, Y., Hu, Y., Zhu, X., Yang, K., Loncar, M.: Octave-spanning Kerr soliton microcombs on thin-film lithium niobate. arXiv preprint arXiv:2403.01107.(2024) 10.1038/s41377-024-01546-7 PMC11369083 39223111

[36] Luke, K., Okawachi, Y., Lamont, M.R., Gaeta, A.L., Lipson, M.: Broadband mid-infrared frequency comb generation in a Si3N4 microresonator. Opt. Lett. **40**(21), 4823–4826 (2015) 26512459 10.1364/OL.40.004823

[37] Moille, G., Westly, D., Srinivasan, K.: Broadband visible wavelength microcomb generation in silicon nitride microrings through air-clad dispersion engineering. arXiv preprint arXiv:2404.01577 (2024)

[38] Coen, S., Randle, H.G., Sylvestre, T., Erkintalo, M.: Modeling of octave-spanning Kerr frequency combs using a generalized mean-field Lugiato-Lefever model. Opt. Lett. **38**(1), 37–39 (2013) 23282830 10.1364/OL.38.000037

[39] Anderson, M.H., Weng, W., Lihachev, G., Tikan, A., Liu, J., Kippenberg, T.J.: Zero dispersion Kerr solitons in optical microresonators. Nat. Commun. Commun. **13**(1), 4764 (2022) 10.1038/s41467-022-31916-x PMC9376110 35963859

[40] Yu, M., Okawachi, Y., Griffith, A.G., Lipson, M., Gaeta, A.L.: Mode-locked mid-infrared frequency combs in a silicon microresonator. Optica **3**(8), 854–860 (2016) 10.1364/OE.24.013044 27410323

[41] Wang, W., Ming, X., Shi, L., Ma, K., Ren, D., Sun, Q., Wang, L., Zhang, W.: Broadband mid-infrared frequency comb generation in a large-cross-section silicon microresonator. IEEE Photonics J. **15**(3), 1–6 (2023)

[42] Zhang, L., Bao, C., Singh, V., Mu, J., Yang, C., Agarwal, A.M., Kimerling, L.C., Michel, J.: Generation of two-cycle pulses and octave-spanning frequency combs in a dispersion-flattened micro-resonator. Opt. Lett. **38**(23), 5122–5125 (2013) 24281525 10.1364/OL.38.005122

[43] Coddington, I., Swann, W.C., Newbury, N.R.: Coherent multiheterodyne spectroscopy using stabilized optical frequency combs. Phys. Rev. Lett. **100**(1), 013902 (2008) 18232764 10.1103/PhysRevLett.100.013902

[44] Bernhardt, B., Ozawa, A., Jacquet, P., Jacquey, M., Kobayashi, Y., Udem, T., Holzwarth, R., Guelachvili, G., Hänsch, T.W., Picqué, N.: Cavity-enhanced dual-comb spectroscopy. Nat. Photonics **4**(1), 55–57 (2010)

[45] Ycas, G., Giorgetta, F.R., Baumann, E., Coddington, I., Herman, D., Diddams, S.A., Newbury, N.R.: High-coherence mid-infrared dual-comb spectroscopy spanning 2.6 to 5.2 μm. Nat. Photonics **12**(4), 202–208 (2018)

